# Histopathological and Toxicological Studies on Zebra Fish Using White-Fruited and Green-Fruited Varieties of *Momordica charantia*

**DOI:** 10.1155/2024/4689625

**Published:** 2024-06-19

**Authors:** Jobi Xavier, Joel Jose, Jayarama Reddy, Paari KA

**Affiliations:** ^1^Department of Life Sciences, Christ University, Bangalore, Karnataka, India; ^2^Department of Botany, St. Joseph's University, Bengaluru, Karnataka, India

## Abstract

*Momordica charantia* is well known for its medicinal properties. It has exhibited various pharmacological activities, such as antidiabetic, anti-inflammatory, and antimicrobial activities. Although this plant is used worldwide as a vegetable and medicinal ingredient in herbal medicines, its toxicity studies have not been conducted to date. This study attempts to understand its toxicity. The present study examined the activity of two enzymes, acetylcholinesterase and succinate dehydrogenase, as well as histopathological variations in the liver, intestine, and gills of zebrafish. The results of the acetylcholinesterase assay showed that the concentrations of 40 mg/L and 60 mg/L of the four extracts (leaf and fruit extracts of both varieties) exhibited increased enzyme activity. Interestingly, the leaves of the green fruit variety at a concentration of 60 mg/L showed the highest activity, with a value of 2.824 ± 0.0682 micromoles/min compared to the control value of 1.8347 ± 0.0046 micromoles/min. On the other hand, the succinate dehydrogenase assay revealed that the concentrations of 40 mg/L and 60 mg/L of the extracts decreased the enzyme activity. The highest inhibition was observed in the concentration of 60 mg/L of the leaves of the white-fruited variety and the green-fruited variety, with values of 1.884 ± 0.0482 micromoles/min compared to the control value of 2.747 ± 0.0046 micromoles/min. The studies on histopathological changes also demonstrated abnormalities in the brain, liver, intestine, and gills of zebrafish after the exposure to the extracts of *M. charantia*. The severity of the damage varied from low to high concentraions. In general, this study sheds light on the safety profile of *Momordica charantia* and highlights its potential toxicity in animal models. The findings suggest that more research is needed to fully understand the toxicity of this plant and its implications for human use.

## 1. Introduction

*Momordica charantia*, commonly known as bitter gourd, has been extensively researched due to its medicinal potential. It belongs to the Cucurbitaceae family and has various pharmacological activities, such as anti-inflammatory, antidiabetic, antimicrobial, and anticancer effects [1]. Two of its powerful ingredients, momordicin and charantin, which are cucurbitane-type triterpenoids, have been linked to its biological actions [2]. Bitter gourd is widely used in South America, Africa, and Asia and is considered safe for human consumption. *M. charantia* has garnered significant attention due to its pharmacobotanical aspects and its effects on various biological systems. In particular, research has spotlighted its pharmacological properties and potential therapeutic benefits, including its impact on glucose metabolism, inflammation, and cancer. However, its safety profile has not been fully evaluated in animal models.

Zebrafish (*Danio rerio*) have been widely used in toxicological and histopathological investigations by the scientific community. Zebrafish are a model organism that reacts to chemicals in a way similar to that of humans and have been used in numerous toxicological and pharmacological research [3]. The safety of natural substances has become a growing concern, and it is crucial to assess how therapeutic herbs impact living things. Therefore, the objective of this study was to assess the toxicity of the white and green *Momordica charantia varieties* in zebrafish and any potential histopathological alterations. *Danio rerio* has been used in numerous investigations as a model organism for behavioral, toxicological, and cancer-related research. Justifications for using this fish as a model organism are its short generation time, external fertilization methods, and reduced maintenance costs for the husbandry. Due to the external nature of fertilization, genetic and developmental modifications are feasible. Since mature zebrafish are small in size, it is possible to assess the cytotoxic effects on the organism and the organ system, which may lead to the development of new metabolites [4].

The main objective of this investigation was to compare the toxicity of the white-fruited bitter gourd (BGW) and green-fruited bitter gourd (BGG) varieties of *M. charantia* in larvae of zebrafish and investigate their histopathological changes. The results of this study will help to understand the safety profile of bitter gourd (*M. charantia*) and develop a better understanding of its toxicity in animal models.

Furthermore, histopathological analysis provides important information on the microscopic modifications caused by these types of bitter gourds. Carefully examining and analyzing these changes can be helpful in identifying possible side effects and understanding the mechanisms underlying the reported toxicological consequences.

The findings of this study will advance our knowledge of *Momordica charantia toxicity* and pave the way for future studies in this field. Thoroughly evaluating the safety profile of this plant using animal models will create a solid foundation for the use of bitter gourd in the development of prospective treatment interventions or dietary guidelines. The results of this study will contribute significantly to a better understanding of *Momordica charantia* and its potential toxicity. This research will provide adequate understanding and guidance for future investigations in the field, ultimately ensuring the safe and effective use of *M. charantia*.

## 2. Materials and Methods

### 2.1. Assay of Acetylcholinesterase Enzyme Activity

#### 2.1.1. Preparation of Plant Extract

The fruits and leaves of the green and white varieties of *M. charantia* (Authentication/SMPU/RARIMD/BNG/2018-19/288) collected from Bangalore, Karnataka were dried and powdered. The powdered plant materials were extracted with methanol. The sample extracts were prepared by adding 50 g of dried powder in 250 mL of methanol using Soxhlet extraction methods. The extract was then subjected to evaporation, and the resulting extract was used for further studies. Serial dilution of *M. charantia* extracts: all four different extracts were diluted to 3 different concentrations (20 mg/mL, 40 mg/L, and 60 mg/mL) [1]. The experiments were carried out using four different extract samples.

#### 2.1.2. Maintenance of *Danio rerio*

The fish were quarantined and stored in a 20 L aquarium and the fish were fed brine shrimp cultured under laboratory conditions once every 36 hours. In total, 13 same-size aquariums were used with one liter of dechlorinated and reverse osmosis water, 12 aquariums were used for testing, and one aquarium for control. Each aquarium was maintained with 3 adult *Danio rerio.*

Administration of plant extracts in fish: the plant extracts were injected into experimental *Danio rerio* using six replicates, including a control following the protocol of OECD [5].

#### 2.1.3. Isolation of Acetylcholinesterase (AChE) from the Brain

The brains of *Danio rerio were* dissected and homogenized with 3 mL of phosphate buffer (PBS) (0.05 M, pH 7.2) using a prechilled mortar and pestle. The homogenate was diluted with 17 mL of cold PB and then centrifuged at 5000 rpm for 15 minutes at 4°C. The supernatant was collected, which served as the source of the enzyme. The enzyme source was diluted at 20x concentration [6].

#### 2.1.4. Acetylcholine Esterase Assay

In this assay [6], to 0.5 mL of enzyme, 5 mL of acetylcholine (100 mM) was added and incubated for 5 minutes at room temperature and then the enzyme was deactivated by placing it in a boiling water bath for 2-3 minutes. The terminated mixture was then titrated against NaOH (0.05 M) with phenolphthalein as an indicator until a pale pink colour was obtained. The titer value was noted, and the enzyme activity was calculated.

### 2.2. Succinate Dehydrogenase (SDH) Assay

#### 2.2.1. Isolation of Mitochondria and Assay of the Succinate Dehydrogenase (SDH) Enzyme

A differential centrifugation technique is adopted to separate mitochondria from a supernatant free of cell debris in brain cells. Osmoprotectants (sucrose or mannitol) are used to help mitochondria stay free of osmotic stress and intact. To prevent heavy metal toxicity, EDTA was used to chelate metal ions and prevent mitochondrial damage. Buffers were used at pH 7.4 or pH 7.2 to maintain the biological pH proximities.

The following reagents were used for the assay of SDH: 1 M sucrose was prepared by adding 342.8 g of sucrose to 1000 mL of distilled water. Tris/HCl buffer was prepared by adding 12.1 g of tris HCl in 500 mL of distilled water, the pH was set at 7.4 and the solution was made up to 1000 mL, 0.1 M EDTA was prepared, and the pH was set at 7.4 using Tris powder. The isolation buffer was prepared by adding 10 mL of 0.1 M Tris/HCl 1 mL of EDTA and 20 mL of 1 M sucrose. Volume was made up to 100 mL and pH was set at 7.4. All prepared reagents were autoclaved at 121°C at 15 PSI for a period of 15−20 min and stored at 4°C. For the extraction of mitochondria, it was essential that the microcentrifuge tube would be fresh and autoclaved. For the performance of the assay, clean test tubes were soaked in 1% H_2_SO_4_. The test tubes were then rinsed in water and dried to remove any water [7].

#### 2.2.2. Isolation of Mitochondria

The tissue was rinsed multiple times using an ice-cold isolation buffer to prevent blood clotting and soaked in the isolation buffer at 4°C and then finely cut the tissue into small pieces. These samples were transferred to a chilled pestle and mortar. The pestle and mortar overnight were prechilled in the freezer and used only while performing the experiment and the tissue was homogenized (powder-like or amorphous) until it became a fine powder (powder-like or amorphous). 5 mL of ice-cold isolation buffer was added for every 1 g of tissue and homogenized until a paste was formed. The homogenate was transferred to a sterile Eppendorf tube and centrifuged at 1600 rpm for 10 min at 4°C. The supernatant was transferred to a sterile Eppendorf tube and centrifuged at 3000 rpm for 15 min at 4°C. The pellet was discarded and the supernatant was transferred to an Eppendorf tube and centrifuged at 7000 rpm for 15 mins at 4°C. The supernatant was discarded and the pellet was resuspended in a fresh isolation buffer, and continued to centrifuge at 10,000 rpm for 15 mins at 4°C. The pellet was stored at 4°C for the enzymatic assay (the pellet was suspended in 0.5 mL of 0.1 M phosphate buffer to use it in the SDH assay). The morphology of the mitochondria was studied by smearing the pellet and staining the smear with Janus green stain. The assay was performed within 24–36 h of isolation [7].

### 2.3. Histological Variations of the Liver, Intestine, and Gills of *Danio rerio*

Histological studies were carried out following the McManus and Mowry protocol [8] and Xavier and Kripasana [9]. After further treatment with *M. charantia* fruit and leaf extracts, intestine, gills, and liver samples were dissected and stored in Bouins fluid for 24 hours. Tissue samples from the control and treatment groups were dehydrated and cleaned in xylene prior to embedding in paraffin wax. Sections of 5 *μ*m thickness were taken and stained with hematoxylin and eosin stains. The sections were mounted in DPX (distyrene plasticizer xylene) after deparaffinization. Sections were observed under a light microscope for structural variations [9].

### 2.4. Statistical Analysis

Statistical analysis of the present study was conducted using SPSS statistical software version 22. The significance of the data was analyzed using Tukey's test at a probability of less than *p* ≤ 0.05.

## 3. Results

### 3.1. Acetycholinesterase Assay

The *in vivo* assay showed that 40 mg/L and 60 mg/L of the four extracts (leaf and fruit extracts of the green and white fruits of *M. charantia*) showed increased enzyme activity. 60 mg/L of leaf extracts from the green-fruited variety of *M. charantia* has shown the highest activity of 2.824 ± 0.0682 micromoles/min when compared to the control of 1.8347 ± 0.0046 micromoles/min. There were no significant differences in the other concentrations of 20 mg/L in the four extracts (Table 1).

### 3.2. *In Vivo Evaluation* of the Succinate Dehydrogenase Activity

In *vivo* assay showed that the 40 mg/L extract and 60 mg/L extract decreased the enzyme activity, and 60 mg of *M. charantia* leaf extracts of the white variety and 60 mg of leaf extracts of the green variety have shown the highest amount of inhibition i.e., 1.884 ± 0.0482 micromoles/min when compared to the control's value of 2.747 ± 0.0046 micromoles/min (Table 2) During the comparison, it was found that the addition of 40 and 60 mg of leaf extracts of both varieties (green and white fruits of *M. charantia*) had a higher rate of inhibition than the fruits.

### 3.3. Histopathological Variations

The general histological evaluation indicated a low to high incidence of tissue damage to *Danio rerio*, after exposure to *M. charantia extracts.* Histopathological changes were observed in the brain, liver, intestine, and gill tissues.

#### 3.3.1. Histopathology of the Brain

**Figure 1:**
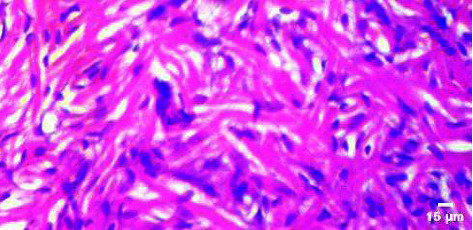
Histopathology of the brain (control).

**Figure 2:**
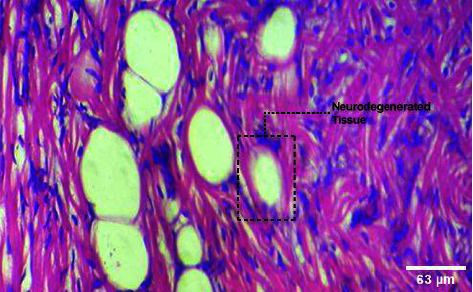
Neurodegeneration with the treatment of 60 mg/mL of leaf extracts of the green-fruited variety (BGG) of *M. charantia*.

**Figure 3:**
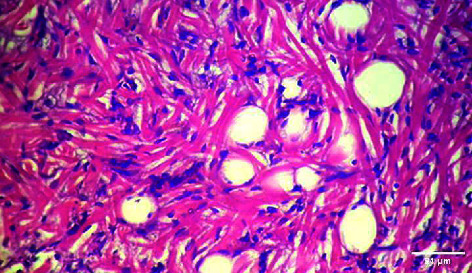
Neurodegeneration with the treatment of 60 mg/mL of leaf extracts of white-fruited variety (BGW) of *M. charantia*.

To determine the histopathological variations of the brain, the fishes were exposed to 40 mg/mL and 60 mg/mL of the plant extracts of *M. charantia* for 4 days. The alteration of the brain tissue was observed under a light microscope (Figures 1, 2, 3). Different concentrations of plant extract treated with fish showed a significant exponential increase in histological modifications over time. In terms of the histomorphology of the brain, obvious tissue chaos was noticed. The brain of *Danio rerio* exposed to a higher concentration of plant extract at 60 mg/mL showed necrosis in the brain. Purkinje cell degeneration was observed in the brain region of zebrafish in the *M. charantia* treatment groups. Disturbed tissue anarchy was observed in the leaf extract-treated samples. Intracellular spaces in the treatment group samples were different from those in the control samples. The presence of serious lesions was observed in the treatment groups, suggesting the toxicity of *M. charantia extracts.*

#### 3.3.2. Histopathology of the Liver Treated with BGG Green Variety Leaf Extract

**Figure 4:**
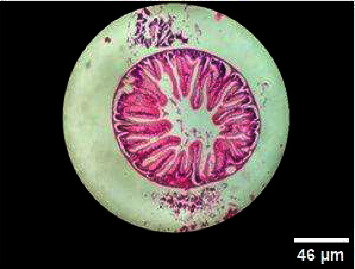
Histopathology of the liver (control).

**Figure 5:**
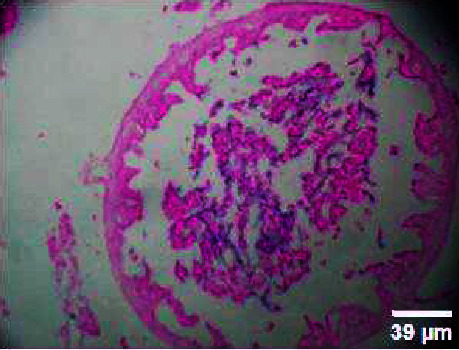
Histopathology of the liver (50 mg/mL).

**Figure 6:**
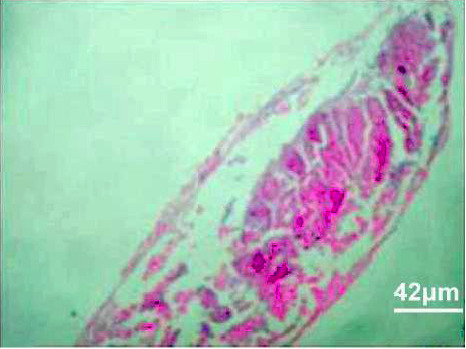
Histopathology of the liver (100 mg/mL).

**Figure 7:**
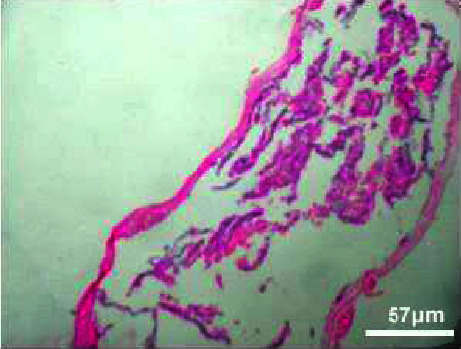
Histopathology of the liver (200 mg/mL).

**Figure 8:**
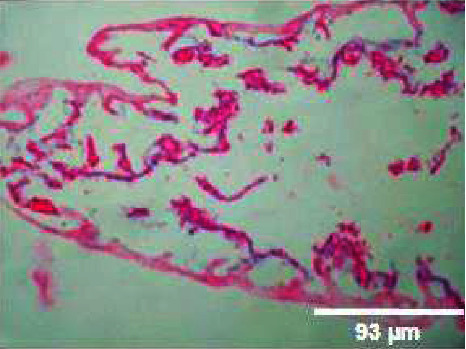
Histopathology of the liver (400 mg/mL).

*M. charantia samples at* higher concentrations of 200 and 400 mg/L have induced nuclear pyknosis and cytoplasmic vacuolization (Figures 4, 5, 6, 7, 8). Hepatic granulation in liver cells seems to be normal in the control samples. The intervals in the hepatic chords were regular. The cytoplasm of the hepatocytes was normal. Erythrocytic infiltration in hepatocytes was found to increase in the treatment groups. Samples at lower concentrations of 50 and 100 mg/L had induced severe cytoplasmic vacuolization.

#### 3.3.3. Histopathology of the Intestinal Tract Treated with Green Variety Leaf Extract (BGG)

**Figure 9:**
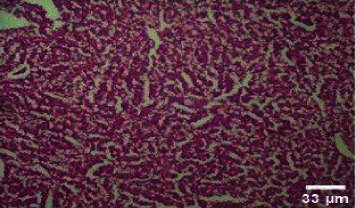
Histopathology of the intestinal tract (control).

**Figure 10:**
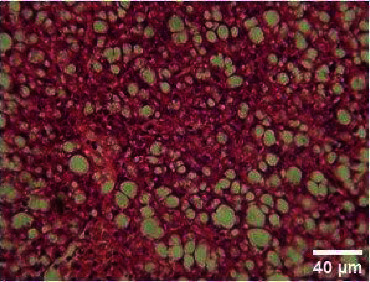
Histopathology of the intestinal tract (50 mg/mL).

**Figure 11:**
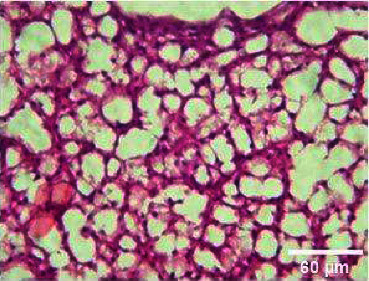
Histopathology of the intestinal tract (100 mg/mL).

**Figure 12:**
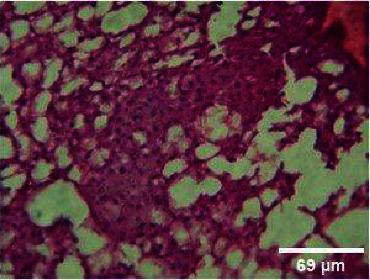
Histopathology of the intestinal tract (200 mg/mL).

**Figure 13:**
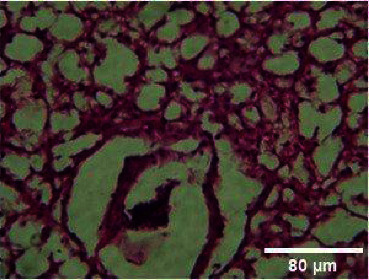
Histopathology of the intestinal tract (400 mg/mL).

Mucosal columnar epithelial cells and goblet cell composition were altered in samples treated with an extract that may influence the function of the intestine. The intestinal villi looked different in the fish exposed to leaf extracts. Columnar epithelial cells were found to be in maximum proportion in all treatment groups and in control samples. There could be damage in mucous-secreting cells that affects the functionality of intestinal cells (Figures 9, 10, 11, 12, 13).

#### 3.3.4. Histopathology of Gills

**Figure 14:**
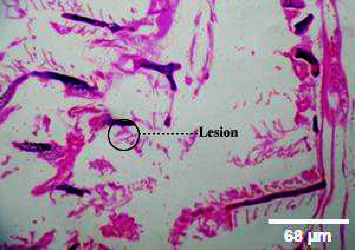
Histopathology of gills (50 mg/mL).

**Figure 15:**
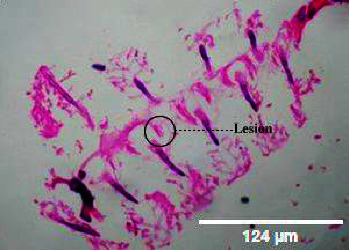
Histopathology of gills (100 mg/mL).

**Figure 16:**
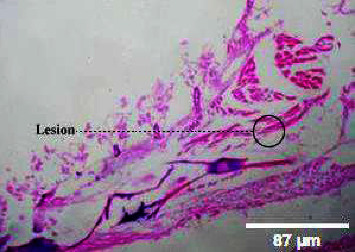
Histopathology of gills (200 mg/mL).

**Figure 17:**
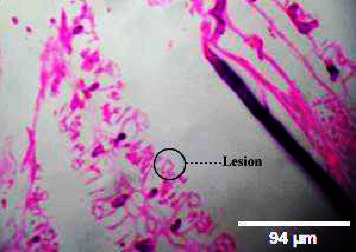
Histopathology of gills (400 mg/mL).

**Figure 18:**
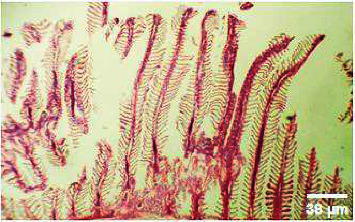
Histopathology of gills (control).

The kind and occurrence of histopathological lesions in fish exposed to different concentrations of M. charantia leaf extracts are shown in the figures. As the gills have a higher chance of reacting to changes in water quality due to *M. charantia* extracts, histopathological studies were carried out in the gill regions to analyze the possible role of *M. charantia* extracts in the regulation of respiration and osmoregulation. However, the severity, but not the type of lesions observed in exposed fish was concentration dependent. Histopathological lesions were observed in the gills of the exposed fish (Figures 14, 15, 16, 17) with increasing concentrations of plant extract. Lesion-like symptoms were observed in the epithelial layer of the cells. The architecture of the primary gill lamellae and the secondary gill lamellae was clearly distinct in the control sample (Figure 18). However, with increasing concentration of extracts, the lamellae were found to curl, causing congestion in the gill structures. This could be another reason for the hemorrhagic conditions observed in the groups of zebrafish treated with different concentrations of the extracts. Changes in the gills of fish exposed to different concentrations of *M. charantia* leaf extracts of *M. charantia* differed significantly from those of nonexposed control fish. Fusion of the tip of the primary lamellae is observed at sample concentrations of 50 mg and 100 mg/L (Figures 14 and 15), whereas detachment of the lamellae is observed at 200 mg/L and 400 mg/L of *M. charantia* Green leaf extract (Figures 16 and 17).

### 3.4. Hemorrhage

**Figure 19:**
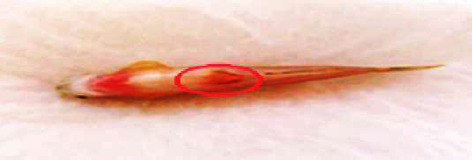
Hemorrhage was observed on the ventral side of the fish.

**Figure 20:**
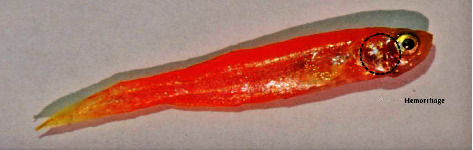
Hemorrhage was observed under the gills of the fish.

**Figure 21:**
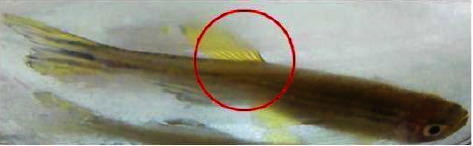
Discoloration on the skin of *Danio rerio* treated with the green leaf extract.

**Figure 22:**
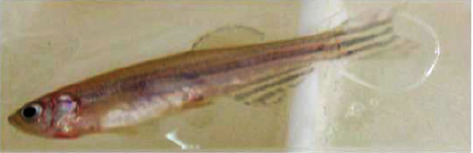
Discoloration on the skin of *Danio rerio* treated with the white leaf extract.

As zebrafish is considered an effective model for vascular development, hemorrhagic conditions for *M. charantia* extracts were examined. The fish suffered from hemorrhage with all 4 higher concentrations. The incidence of hemorrhage might be due to rupture of the blood vessels. This was subjected twice, for two different healthy *Danio rerio*, but resulted in the presence of blood spots near the ventral region, especially near the fins and jaw. Signs of hemorrhage with restlessness were also confirmed in zebrafish treated with the extracts. Leakage or damage to blood vessels might be due to ruptured blood vessels. It was also observed that these fish were unable to regain their health immediately after the withdrawal of the extracts; however, a slight discoloration was observed in a few samples. The integrity of the vessel was disturbed and a defect in the vascular wall was observed on both the ventral sides of the fish and beneath the gill regions (Figures 19 and 20). The marked changes in tissue discoloration may be due to changes in the architecture of endothelial cells that form the barrier between blood and surrounding tissue (Figures 21 and 22). The results of the study indicate that the architecture is disturbed due to the presence of extracts from *M. charantia.*

## 4. Discussion

### 4.1. Acetylcholinesterase (AChE) Enzyme Assay

Acetylcholinesterase (AChE) is one of the most effective enzymes in the nervous system. It is intensely concentrated at the cholinergic synapses. Acetylcholinesterase has been associated with certain neuromuscular disorders such as glaucoma, myasthenia gravis, and most recently Alzheimer's disease. Clinically, moderate inhibition of acetylcholinesterase is effective in the treatment of Alzheimer's disease or reduced expression of fewer receptors, as in the case of myasthenia gravis, it is a possible way to tackle this disease [6]. The increased amount of acetylcholinesterase may cause different neurological disorders due to overexpression and thus reduce the amount of acetylcholine in the cholinergic synapses.

This may lead to lesions in the nervous systems. Acetylcholinesterase is involved in various metabolic activities of the organism that include cell cycle regulation and processes related to Alzheimer's disease. However, more studies need to be conducted to prove the direct relationship between the increase in AChE and nervous disorders. The increased concentration of acetylcholine esterase in tissues indirectly reflects a reduced concentration of acetylcholine that causes local and systemic inflammation.

When the increase in acetylcholinesterase concentrations was observed, acetylcholine levels decreased, and this can lead to a reduction in the anti-inflammatory properties exercised by acetylcholine. Thus, an increase in the concentration of acetylcholine esterase in plasma CSF, leukocytes, RBC, platelets, and other tissues indirectly affects the reduced concentration of acetylcholine and the increase in local and systemic inflammation. This inflammation can be attributed to the toxic contribution of the *M. charantia* extracts to zebrafish. According to Marroquín-Segura et al., increased acetylcholinesterase activity in plasma, RBC, CSF, leukocytes, platelets, and other tissues can be considered a reliable source for identifying acute, chronic, and low-grade systemic inflammation [10].

### 4.2. Succinate Dehydrogenase Enzyme

Studies by Rustin et al. have revealed that inherited defects of mitochondrial succinate dehydrogenase in humans are associated with striking variable clinical presentations ranging from the early onset of encephalomyopathy to susceptibility to tumors in adulthood [7]. Inactivation of the enzyme succinate dehydrogenase can lead to accumulation of succinate in the mitochondria causing leakage to the cytosol. This can result in the inhibition of a group of prolyl hydroxylase enzymes (PHDs). Depending on the inhibition of PHD, two newly recognized pathways that support tumourigenic effects can become functional, that is, affected cells become resistant to certain apoptotic signals and/or activate a pseudohypoxic response that enhances glycolysis and is conveyed by hypoxia-inducible factor [11].

Previous studies by others also support the view that inhibition of SDH will result in tumorigenesis in animals and humans [12]. The same view was supported by the present study conducted in *Danio rerio* with extracts of the *M. charantia* plant. In this study, it was clearly proved that higher concentrations of BGG and BGW plant extracts have decreased the activity of SDH enzymes, thus causing malfunctions in the development of *Danio rerio.*

The plant extracts of *Momordica charantia,* although considered to have medicinal properties, were found to have toxic effects when used in higher concentrations. An attempt was made to analyze the toxic levels of plant extracts in the present study. It was clear from the experiments that *M. charantia plant* extracts were found to be safe up to the dose of 40 mg/kg body weight. The inactivation of SDH activity was found to be crucial at 60 mg/kg, which could lead to tumor development at later stages. Therefore, it is safe to control the use of *M. charantia leaves.* Controlling the use of the leaves of the plant will help prevent different medical complications caused by the use of *M. charantia.*

### 4.3. Histopathological Variations

*Danio rerio* exposed to a sublethal concentration of *M. charantia* extract showed significant adverse changes in the liver, intestinal tract, brain, and gills. The liver is the main storage organ of lipids and the site of metabolic activities in fish that helps in detoxification. The organ is also associated with the biotransformation process. However, when the liver was exposed to *M. charantia* plant extracts, it showed hepatocyte degeneration characterized by cytoplasmic vacuolization. Exposure to the extract resulted in the appearance of a large number of hepatocytes with pyknotic nuclei and hemorrhage surrounding the central vein and severe necrosis in the liver. The results of our study could be correlated with previous studies conducted in *Danio rerio* exposed to sublethal concentrations of pesticides [13].

*M. charantia* is used as an alternative medicine that has been used primarily to lower blood glucose levels in patients with diabetes mellitus. Studies have revealed that the components of the *M. charantia* extract appear to have structural similarities to animal insulin. Clinical trials found that *M. charantia* extracts have a moderate hypoglycemic effect. Although the extracts of *M. charantia* fruits were found to have moderate hypoglycemic effects in many of the studies conducted, the early effects of plant fruit extracts have not been thoroughly studied. Some studies have reported the advanced effects of the hypoglycemic compound convulsion of *M. charantia* in children, reduced fertility in mice, a favism-like syndrome, increased gamma-glutamyl transferase and alkaline phosphatase levels in animals, and headaches [14]. Since there were no studies conducted on the toxicology of the plant extracts of *M. charantia* that are widely used by the common people, the present study was an attempt to understand the toxic levels of the plant extracts from leaves and fruits of *M. charantia.*

Fish have the ability to adapt to changes in their environment by increasing the distance of diffusion to their blood. They do it by forming a barrier through proliferating epithelial cells and fusion of primary and secondary lamellar cells [15]. However, when the gills of catfish (*Clarias gariepinus*) were exposed to extracts of *Parkia biglobosa*, fruits showed the appearance of lesions [16] and, in some cases, epithelia proliferation in response to epithelial damage [17]. Functional loss or morphological damage was observed in affected gill structures of fish exposed to various toxic elements [18–20].

### 4.4. Hemorrhage

In the present study, hemorrhage was observed on the ventral side of zebrafish treated with a 60 mg/mL plant extract of *M. charantia*. This hemorrhage was caused by damage to the pillar cells and the subsequent loss of their supportive properties. Similar findings were reported in a previous study by Camargo and Martinez [21]. As a result of this damage, blood cells leaked into the epithelial layer, which could have affected the free flow of blood and subsequently caused an impairment of gaseous and ionic exchange in fish. Furthermore, recovery from gill damage was found to be slow and difficult. An increase in the cellular layers of the lamellar epithelium could be attributed to an increase in the mitotic divisions of the lamellar epithelium. According to Kantham and Richards [22], this increase in epithelial thickness is a natural response to retard the bloodstream. In addition, we observed lamellar fusion as a result of the thickening of the gill filament epithelium. The fusion and hyperplasia of gill lamellae could have been caused by toxins present in the *M. charantia* extract, which modify the glycoproteins present in the mucus that cover the cells and alter the negative charge of the epithelium, thus favoring adhesion to adjacent lamellae. This conclusion is consistent with earlier reports by Ferguson [23].

## 5. Conclusions

The present study aimed to evaluate the toxicity of two varieties of *Momordica charantia* (bitter gourd) in zebrafish larvae and investigate their histopathological changes. The study found that the extracts of both the green and white-fruited varieties of *Momordica charantia* exhibited significant effects on the enzyme activity of acetylcholinesterase (AChE) and succinate dehydrogenase (SDH) in zebrafish. The AChE activity increased with higher concentrations of the extracts, while the SDH activity decreased. Furthermore, histological analysis revealed structural variations in the liver, intestine, and gills of zebrafish after treatment with the extracts. These findings contribute to understanding the safety profile of *Momordica charantia* and highlight its potential toxicity in animal models. Further research is warranted to explore the underlying mechanisms and potential therapeutic applications of this medicinal plant.

## Figures and Tables

**Table 1:** Acetylcholinesterase (AChE) activity with different concentrations of the extract.

Samples (mg/mL concentration)	Enzyme activity (*μ*mol/min)
Control	1.835 ± 0.0046^a^
40 mg of sample-L1	2.543 ± 0.0046^b^
60 mg of sample-L1	2.824 ± 0.0682^c^
40 mg of sample-L2	2.343 ± 0.0046^b^
60 mg of sample-L2	2.613 ± 0.0046^c^
40 mg of sample-F2	2.613 ± 0.0046^b^
60 mg of sample-F2	2.743 ± 0.0646^c^
40 mg of sample-F1	2.491 ± 0.0046^b^
60 mg of sample-F1	2.813 ± 0.0446^c^

L1, leaf extract of green variety; L2, leaf extract of white variety; F1, fruit extract of green variety; F2, fruit extract of white variety. Results are means of triplicates (mean ± SD). The various superscript letters indicated significant differences between the samples at *p* ≤ 0.05.

**Table 2:** Succinate dehydrogenase (SDH) activity with different concentrations of the extract.

Samples	Enzyme activity of *M. charantia* leaves and fruits			
	Leaf extracts of the white variety of *M. charantia*	Leaf extracts of the green variety *M. charantia*	Fruit extracts of the white variety of *M. charantia*	Fruit extracts of the green variety of *M. charantia*
Control	2.747 ± 0.0046^a^ micromoles/min			
40 mg/L	2.142 ± 0.0046^b^ micromoles/min	2.243 ± 0.0046^b^ micromoles/min	2.291 ± 0.0046^b^ micromoles/min	2.143 ± 0.0046^b^ micromoles/min
60 mg/L	1.884 ± 0.0482^c^ micromoles/min	1.884 ± 0.0482^c^ micromoles/min	2.013 ± 0.0046^c^ micromoles/min	1.943 ± 0.0046^c^ micromoles/min

Results are means of triplicates (mean ± SD). The various superscript letters indicate that there was a significant difference between the samples at 0.05.

## Data Availability

The data used to support the findings of this study are available from the corresponding author upon request.
